# Phenotypic Behavioural Effects of Genetic Deletion of the Vesicular Glutamate Transporter 3 in 5‐Hydroxytryptamine Neurons

**DOI:** 10.1111/gbb.70063

**Published:** 2026-07-27

**Authors:** L. Sophie Gullino, Nida Chabbah, Spatika Jayaram, Raquel Pinacho, Salah El Mestikawy, Stéphanie Daumas, David M. Bannerman, Trevor Sharp

**Affiliations:** ^1^ Department of Pharmacology University of Oxford Oxford UK; ^2^ Sorbonne Université INSERM, CNRS, Centre for Neuroscience (NeuroSU)– Institut de Biologie Paris Seine (IBPS) Paris France; ^3^ Department of Experimental Psychology University of Oxford Oxford UK; ^4^ Douglas Mental Health University Institute, Department of Psychiatry McGill University Montreal QC Canada

**Keywords:** 5‐HT, anxiety, glutamate, learning, reward, serotonin, VGLUT3

## Abstract

A major subpopulation of 5‐hydroxytryptamine (5‐HT) neurons expresses the vesicular glutamate transporter 3 (VGLUT3) allowing the co‐release of glutamate. Previous evidence has implicated VGLUT3 in 5‐HT neurons in mechanisms of anxiety and reward. Here we examined mice with a genetic loss of VGLUT3 targeted to 5‐HT neurons (VGLUT3 cKO^5‐HT^) and littermate controls in a battery of behavioural tests, including paradigms assessing levels of anxiety and learning with appetitive rewards. Compared to littermate controls, VGLUT3 cKO^5‐HT^ mice displayed no evidence of altered anxiety‐like behaviour in the elevated plus maze, light/dark box, marble burying and social interaction tests. However, VGLUT3 cKO^5‐HT^ mice showed reduced preference for low (but not high) sucrose‐containing solution and reduced correct responses in an appetitively motivated spatial reference memory task. Similarly, in an appetitively motivated operant task, VGLUT3 cKO^5‐HT^ mice displayed evidence of reduced responding to cues associated with reward. These effects appeared specific in that VGLUT3 cKO^5‐HT^ mice did not differ from controls in terms of home cage food consumption, performance in a spatial novelty preference test, as well as contextual and cued fear memory tests. These findings support a role for VGLUT3 in 5‐HT neurons in some aspects of learning, here in association with learning for reward, although not anxiety‐like behaviour.

## Graphical abstract

VGLUT3 in 5‐HT neurons is involved in some aspects of learning, here in association with learning for reward, although not anxiety‐like behaviour.

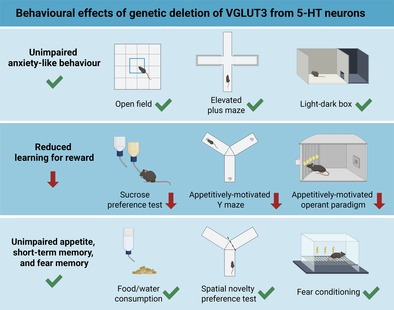

**Abbreviations**
5‐HT: 5‐hydroxytryptamine
ARRIVE: animal research: reporting of in vivo experiments
cKO: conditional knockout
CS: conditioned stimulus
DRN: dorsal raphe nucleus
EPM: elevated plus maze
GABA: gamma‐aminobutyric acid
ITI: inter‐trial interval
SEM: standard error of the mean
SERT: serotonin transporter
US: unconditioned stimulus
VGLUT3: vesicular glutamate transporter 3
VTA: ventral tegmental area

## 1 Introduction

A major subpopulation of midbrain 5‐hydroxytryptamine (5‐HT) neurons expresses the Type 3 vesicular glutamate transporter (VGLUT3) enabling the co‐release of glutamate as well as vesicular accumulation of 5‐HT [1, 2, 3, 4, 5, 6, 7, 8]. Evidence of 5‐HT‐glutamate co‐release was first identified by electrophysiological recordings in cultured rat 5‐HT neurons [9]. VGLUT3 gene expression was then discovered in midbrain 5‐HT neurons, including approximately two‐thirds of 5‐HT neurons in the dorsal raphe nucleus (DRN) [2, 4, 5, 10]. Subsequently, the co‐release of 5‐HT and glutamate was demonstrated in various forebrain regions using a combination of 5‐HT neuron‐targeted optogenetics and electrophysiology [11, 12, 13]. Currently, the behavioural impact of VGLUT3 expressed in 5‐HT neurons is poorly understood.

Clues about the function of VGLUT3 expression in 5‐HT neurons have come from behavioural studies of mice with global genetic deletion of VGLUT3 [8, 14, 15, 16]. Thus, adult VGLUT3 knockout (KO) mice demonstrated increased anxiety‐like behaviour on the elevated plus maze (EPM), novelty suppressed feeding test and marble burying, while VGLUT3 KO pups displayed increased ultrasonic vocalisation [8]. These VGLUT3 KO mice also showed increased contextual fear learning and fear generalisation, as evidenced by exaggerated freezing during fear conditioning [15, 17]. In addition, VGLUT3 KO mice displayed mild learning deficits, including reduced performance in tests of avoidance‐based learning (e.g., the ‘shuttle‐box’), as well as impaired reversal learning in a reward‐based operant paradigm [16].

Interpretation of these data is complicated because VGLUT3 is expressed not only in midbrain 5‐HT neurons but also in other neuron types including cortical and hippocampal gamma‐aminobutyric acid (GABA) interneurons, striatal cholinergic interneurons and subpopulations of glutamatergic neurons [5, 7, 18]. Nevertheless, given the well‐established links between 5‐HT and anxiety [19, 20, 21], the high anxiety phenotype of VGLUT3 KO mice was considered driven by the lack of VGLUT3 in 5‐HT neurons [8]. However, a more recent study [22] found that mice with VGLUT3 KO targeted to neurons expressing the 5‐HT regulator gene Pet1 were hypoactive in the open field and EPM, but did not display decreased time spent in the open arms which is often seen as a sign of anxiety. Thus, the latter finding calls into question the proposed role of VGLUT3 expressed in 5‐HT neurons in anxiety.

Interestingly, several lines of evidence suggest that VGLUT3‐dependent glutamate co‐released from 5‐HT neurons may play a role in learning for reward. Thus, in vitro electrophysiological studies found that optogenetic activation of DRN VGLUT3‐expressing 5‐HT neurons excited mesolimbic dopaminergic neurons in the ventral tegmental area (VTA), which are well‐known to play a key role in reward processing [22, 23]. Furthermore, in vivo optogenetic activation of DRN 5‐HT neurons or their VTA terminals elicited place preference [11, 23] and these rewarding effects were abolished by combined VGLUT3 and 5‐HT depletion [11]. Nonetheless, other optogenetic studies have found no rewarding effect of DRN 5‐HT neuron activation in terms of place preference [24] and additional data indicate that DRN VGLUT3‐expressing neurons that lack 5‐HT also exert rewarding effects [25, 26].

Clarification of the behavioural role of VGLUT3 in 5‐HT neurons is translationally relevant as VGLUT3 expression is likely to vary in the human population, with point mutations of the gene encoding VGLUT3 (Slc17a8) resulting in life‐long alterations in VGLUT3 expression [27]. Additionally, 5 HT neurons can likely alter their co‐release profile through changes in VGLUT3 expression due to environmental triggers such as chronic stress [28, 29].

Here, we investigated the behavioural phenotype of a transgenic mouse with genetic loss of VGLUT3 targeted to 5‐HT neurons (VGLUT3 cKO^5‐HT^), which we have previously validated [30, 31]. VGLUT3 cKO^5‐HT^ mice underwent a battery of behavioural tests of anxiety‐like behaviour, anhedonia and appetitively motivated learning, as well as tests probing non‐appetitive learning and memory, including cued and contextual fear conditioning.

## 2 Material and Methods

### 2.1 Animals

Transgenic mice with conditional VGLUT3 KO in 5‐HT neurons (VGLUT3 cKO^5‐HT^ or SERT‐Cre::VGLUT3^LoxP/LoxP^, C57BL/*6J background, aged* 8–30 weeks) and their control littermates (SERT^+/+^::VGLUT3^LoxP/LoxP^) were used. VGLUT3 cKO^5‐HT^ mice were originally generated by crossing mice which expressed Cre recombinase under the control of the SERT (serotonin transporter) promoter (SERT‐Cre) with floxed VGLUT3 mice (VGLUT3^loxP/loxP^) [30, 31, 32] (Figure S1). For the experiments described here, mice were bred by crossing SERT‐Cre::VGLUT3^LoxP/LoxP^ mice with SERT^+/+^::VGLUT3^LoxP/LoxP^ mice (Figure S1; as in [30]), thus littermate controls also expressed floxed VGLUT3 but were wildtype for SERT. As there are reports that Cre recombinase expression itself can alter behaviour [33, 34, 35], certain experiments also compared SERT‐Cre mice with wildtype (SERT^+/+^) controls to examine whether Cre expression in 5‐HT neurons might produce phenotypic changes.

VGLUT3 conditional heterozygous^5‐HT^ mice (SERT‐Cre::VGLUT3^LoxP/+^) were generated and examined in an initial pilot study, but this preliminary analysis did not reveal evidence of a phenotype. Consequently, all subsequent studies utilised homozygous mice.

Mice were group‐housed (2–6 per cage) with littermates in individually ventilated cages in a temperature‐controlled room (21°C) with a 12 h light/dark cycle (light on 07:00, lights off 19:00). Mice had ad libitum access to food and water (unless otherwise specified) and cages were lined with sawdust bedding and contained cage enrichment consisting of sizzle nest and a cardboard tube. To minimise stress, mice were habituated to handling on three occasions before any experiment and handling was always carried out using a cardboard tunnel [36, 37].

Experiments followed the principles of the Animal Research: Reporting of In Vivo Experiments (ARRIVE) guidelines and were conducted according to the UK Animals (Scientific Procedures) Act of 1986, with appropriate personal and project licence coverage. A subset of experiments (see Cohort 5 below) was conducted in France, in accordance with the European Communities Council Directive for the Care and the Use of Laboratory Animals and in compliance with the French Ministère de l'Agriculture et de la Forêt, Service Vétérinaire de la Santé et de la Protection Animale. All measurements were obtained and analysed by a researcher blind to genotype.

### 2.2 Behavioural Tests

**FIGURE 1:**
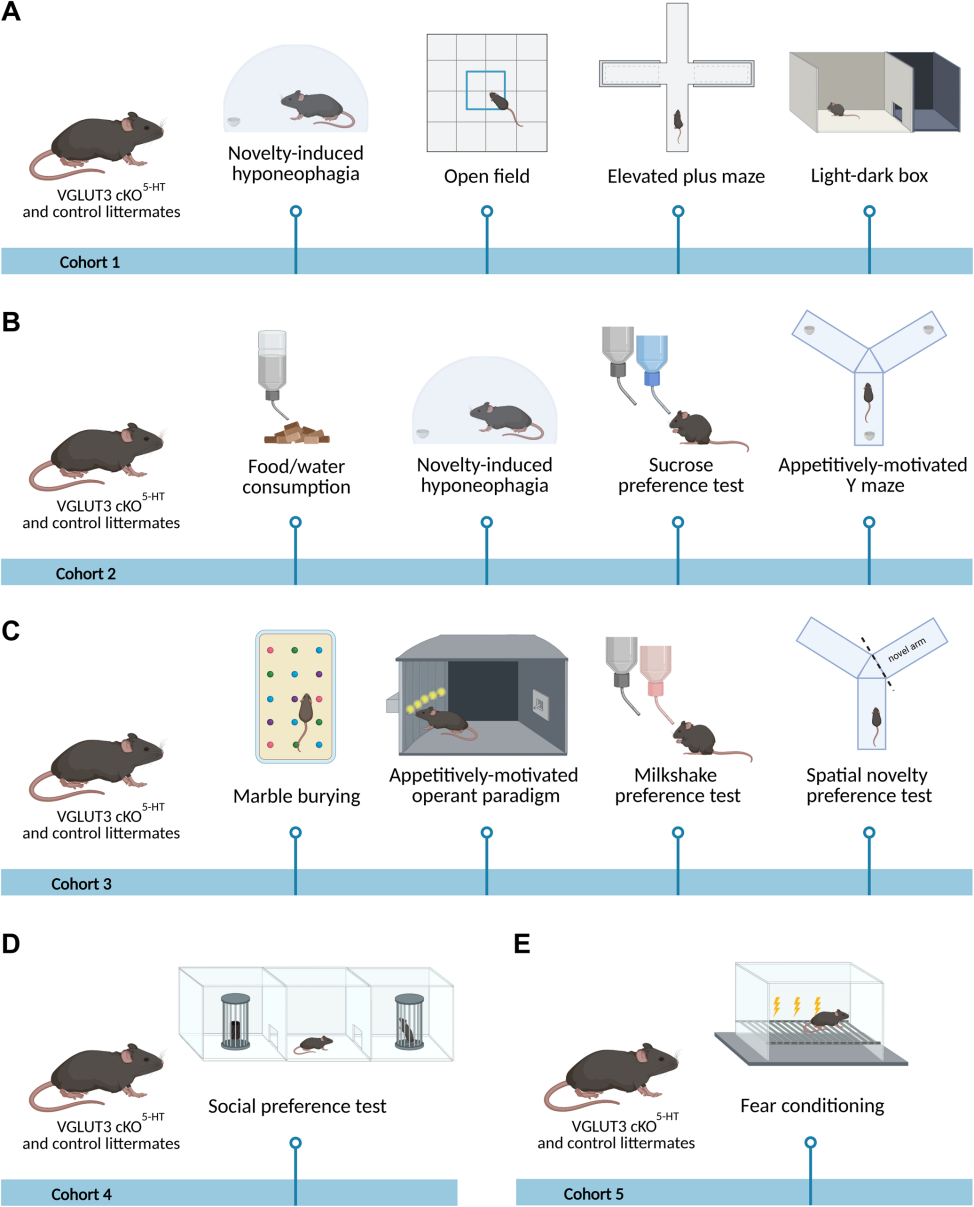
Experimental timelines of behavioural experiments. Experimental timelines of (A) cohort 1 using tests of anxiety‐like behaviour, (B) Cohort 2 using appetitively motivated tests of anxiety, anhedonia and reward, (C) Cohort 3 using an appetitively motivated operant learning paradigm and tests of short‐term habituation, (D) Cohort 4 and (E) Cohort 5.

VGLUT3 cKO^5 HT^ mice and control littermates were randomly assigned to one of five cohorts which were examined in a battery of different but complementary tests. Sample size was based on pilot experiments. Cohort 1 (cKO^5‐HT^ *n* = 13, control *n* = 12) was assessed for anxiety‐like behaviour and locomotion using the novelty‐induced hyponeophagia test, non‐anxiogenic open field, EPM and light/dark box (Figure 1A). Cohort 2 was examined for general food/water consumption and body weight (cKO^5‐HT^ *n* = 36, control *n* = 36) as well as in the novelty‐induced hyponeophagia test (cKO^5‐HT^ *n* = 13, control *n* = 13), sucrose preference test (cKO^5‐HT^ *n* = 18, control *n* = 20) and an appetitively motivated spatial reference memory paradigm (cKO^5‐HT^ *n* = 17, control *n* = 16) (Figure 1B). Cohort 3 was assessed for marble burying (cKO^5‐HT^ *n* = 13, control *n* = 13) as well as performance in a food‐restricted appetitively motivated operant paradigm, milkshake preference test (cKO^5‐HT^ *n* = 15, control *n* = 17) and a spatial novelty preference test (cKO^5‐HT^ *n* = 17, control *n* = 16) (Figure 1C). Cohort 4 (cKO^5‐HT^ *n* = 12, control *n* = 13) was examined using a social preference test (Figure 1D). Finally, cohort 5 (cKO^5‐HT^ *n* = 16, control *n* = 15) was tested for cued and contextual fear conditioning (Figure 1E). All cohorts were mixed sex except Cohort 5, which was male. Litter size ranged from 1 to 12 mice, but averaging around 6 mice per litter so that each experiment utilised a minimum of 4 litters. A cohort of SERT‐Cre mice and wildtype controls was also assessed using the non‐anxiogenic open field, EPM, sucrose preference test as well in the appetitively motivated operant paradigm.

Test batteries always involved at least 2 days of rest in between each test. All experiments were conducted during the light phase (10:00–17:00) unless otherwise specified. Mice were always allowed to habituate to the testing room for at least 30 min prior to experimentation. In all experiments involving an apparatus without bedding, the apparatus was wiped between animals with 0.5% Anistel (Tristel) to remove odours.

#### 2.2.1 Non‐Anxiogenic Open Field

To test for differences in levels of locomotion and exploratory activity, a non‐anxiogenic open field was used as previously described [38]. The apparatus consisted of a square arena (50 × 50 cm) located in a dimly lit room. Mice were placed in the outer zone (close to the walls) and were free to explore the arena for 10 min. Locomotor activity was recorded with an overhead camera and ANY‐maze software (Stoelting Europe) was used for offline measurement of total distance travelled, time spent in the outer and centre zones (15 cm square) and latency to enter the centre zone.

#### 2.2.2 EPM

The EPM was used to assess unconditioned anxiety as previously described [39, 40]. The apparatus comprised a cross‐shaped elevated maze (70 cm height) with two open arms (35 × 6 cm) perpendicular to two closed arms (35 × 6 cm, 20 cm walls) located in a dimly lit room [39]. Mice were placed in the closed arm (counterbalanced between the two closed arms) and allowed to explore for 5 min. Movement on the EPM was monitored using an overhead camera and ANY‐maze software (Stoelting Europe) was used for offline measurement of time spent, latency to enter and number of entries into the open arms. Total distance travelled was also measured.

#### 2.2.3 Light/Dark Box

The light/dark box was carried out as previously described [41, 42] using a wooden box comprising a black covered compartment (21 × 16 cm, 16 cm walls) with a small doorway leading to a white and brightly lit open compartment (21 × 16 cm, 16 cm walls). Mice were placed into the dark compartment and allowed to explore for 10 min, during which their movements were monitored via an overhead camera. Total entries, latency to enter and time spent in the light compartment were manually scored offline.

#### 2.2.4 Marble Burying

The marble burying test was selected as previous evidence showed that mice with global VGLUT3 KO buried marbles faster than controls, which was considered evidence of neophobia [8]. The marble burying test was carried out using a clean cage (14 × 32 × 13 cm) containing a 5 cm layer of flattened sawdust bedding and 12 colourful glass marbles (20 mm diameter), which were positioned on the surface of the sawdust, evenly spaced. Mice were individually placed in the cage for 30 min and burying activity was recorded by an overhead camera. Latency to start digging anywhere in the cage and number of marbles buried (in 3 min time bins) were obtained from the video recordings. A marble was considered buried when over two‐thirds of its surface was covered in sawdust, as previously described [8, 43].

#### 2.2.5 Social Preference Test

The social preference test was carried out over two consecutive days (adapted from [44]) to assess preference for an unfamiliar conspecific compared to an inanimate object, with reduced social preference suggesting increased anxiety‐like behaviour [45]. The test setup consisted of a rectangular box (made of red see‐through Plexiglas) comprising three connected consecutive chambers (19 × 45 cm). The two side chambers contained two barred cages (7 × 7 cm). On the first day of the test, the barred cages were empty and mice were individually habituated to the three‐chamber box for 10 min. On the testing day an unfamiliar mouse (of the same‐sex and age‐matched to the experimental mouse) was placed in a barred cage in one of the side chambers, whilst the cage located in the opposite side chamber contained a novel object. Positions of the unfamiliar mouse and the novel object were counterbalanced between groups. Mice were allowed to explore all three compartments for 10 min. Exploration of the compartment with the unfamiliar mouse was used as a measure of social preference.

#### 2.2.6 Food/Water Consumption

Food and water consumption were assessed as changes in these measures could affect performance in appetitively motivated tests. Mice were single‐housed overnight (17:00–9:00) in individually ventilated cages (14 × 32 × 13 cm), with ad libitum water and 7 g of regular chow (which was familiar to them). Cages were lined with sawdust bedding and contained a square of compressed cotton (‘Nestlet’, Datesand). Food and water consumption were recorded over two nights and measurements were averaged.

#### 2.2.7 Novelty‐Induced Hyponeophagia

Mice were exposed to the novelty‐induced hyponeophagia as this is a well‐established test of anxiety with an appetitive component [46, 47]. For novelty‐induced hyponeophagia, mice were food deprived overnight and tested the following day (~18 h later) as previously described [48, 49]. Mice were weighed prior to testing to ensure that their body weight had not fallen below 90%. The testing apparatus consisted of a white plastic arena covered with an upturned transparent jug (15 cm diameter) with a spout protruding 2 cm to a food well. The well was filled with sweetened condensed milk (50% water), which was novel to the mice. Mice were tested across a maximum of three 120 s trials, during which latency to approach the novel food and latency to continuously drink were manually recorded. In between each trial, mice were placed in a clean cage. The trial ended once drinking commenced and no further trials took place. If a mouse did not drink during the three trials, the latency to drink was recorded as 360 s.

#### 2.2.8 Sucrose Preference Test

The sucrose preference test is typically used to assess anhedonia, which has been defined as reduced ability to experience pleasure from a rewarding stimulus, as well as to detect differences in reward sensitivity [50]. The sucrose preference test was conducted in the dark phase (17:30–1:00) over four consecutive evenings, as previously described [51]. For the first two evenings (3 h each) mice were single‐housed and habituated to the testing setup which consisted of an open top cage (42 × 22 cm, 20 cm walls) lined with bedding, containing two water bottles and food available *ad libitum*. On the third evening (2 h) mice were offered 2.5% sucrose in a single bottle to reduce neophobia for the sucrose solution during the testing day. The testing took place on the fourth evening (7 h) when the mice were presented with one bottle containing water and another one containing 1% sucrose. This concentration was chosen as decreased preference for sucrose can be reliably detected using 1%–2% sucrose solutions but not higher concentrations [50, 52]. On the third and fourth evenings the positions of the bottles were swapped half‐way through the session to reduce the influence of any side preference. Bottles were weighted before and after the test to measure water and 1% sucrose consumption. Preference score was calculated as sucrose solution consumption minus water consumption [53].

#### 2.2.9 Appetitively Motivated Spatial Reference Memory Test

An appetitively motivated, spatial reference memory test was carried out over 11 days [48]. This task is a form of conditioned place preference, a test which has been previously implicated in the functions of 5‐HT‐glutamate co‐release [11, 23]. From the day before testing to the end of the experiment mice were food restricted to 90% of their free‐feeding body weight. The apparatus consisted of a wooden elevated Y‐maze (50 × 9 cm arms with food well at the end, 0.5 cm walls) placed in a room with a variety of extra‐maze cues. Before testing each mouse was assigned a ‘target’ arm which was rewarded with sweetened condensed milk (50% water) throughout the testing. Identification of the ‘target’ arm required mice to use allocentric extra‐maze cues as opposed to intra‐maze cues. Mice underwent one habituation day (free maze exploration) and then 10 training days with 10 trials per day. On each trial mice were placed on the start arm farthest from the centre of the maze and allowed to approach the centre area and enter an arm of their choice. Choice of the target arm was considered a correct response. Conversely, an incorrect response was registered if the target arm was not selected. On completion of the trial mice were removed from the apparatus and single‐housed in clean cages between trials. On the final day of testing, to ensure that the reward itself was not serving as an olfactory cue, the target arm was baited with reward only after the mice had reached the selected arm (i.e., post‐choice baiting). The starting arm (i.e., one of the two unbaited arms) was assigned pseudo‐randomly for each trial but was counterbalanced between groups. The target arm was also counterbalanced across groups but remained constant for an individual mouse across all trials and the maze was rotated 120° every few trials to avoid the use of intra‐maze cues. The daily % of correct arm entries were recorded for each mouse.

#### 2.2.10 Appetitively Motivated Operant Paradigm

The appetitively motivated operant paradigm was used to further investigate learning with reward in VGLUT3 cKO^5‐HT^ mice. Five days prior to commencing the appetitively motivated operant paradigm, mice were food restricted and remained so throughout the experiment (maintained at about 90% of free‐feeding body weight). Mice were habituated to the reward (strawberry milkshake; Yazoo kids) in their home cages to reduce subsequent hyponeophagia in the operant chambers. Operant boxes (20 × 20 cm, Med Associates, Figure S2A) were placed in sound‐attenuated dark chambers and were equipped with a panel of five nose‐poke ports with recessed LED lights. A milkshake ‘magazine’ was located on the wall opposite to these ports (Figure S2A); this comprised a drinking spout which dispensed milkshake (~12 μL) via a syringe pump. Milkshake delivery and illumination of nose‐poke ports were controlled by Med‐PC software package (Med Associates, SOF‐735). No light cue was used to indicate reward availability.

On the first day of training, mice were habituated to the operant box for 30 min, during which five milkshake administrations were delivered (once every 5 min) from the magazine (Table S1). At this stage, no ports were illuminated and no action was required to trigger milkshake delivery. The number of magazine head entries and nose‐pokes into the ports was recorded.

Over the following 7 days mice were trained to perform an appetitively motivated operant task in which a nose‐poke into any of five illuminated ports triggered delivery of a milkshake reward from the magazine on the opposite wall (Table S1, Figure S2A). This task has been previously used as a habituation stage for the five‐choice serial reaction time task [54] and involved all five ports being illuminated simultaneously. Each training session started with a milkshake delivery (not contingent on any action) and its consumption triggered an inter‐trial interval (ITI) of 2 s, after which all five ports were illuminated until the mouse nose‐poked a port. This was recorded as a correct response and triggered delivery of a milkshake reward (Figure S2B). Each day mice were tested for 30 min or until 40 correct trials were reached. The number of correct responses, magazine head entries, latency to respond to the light stimulus with a nose‐poke (timed from when the lights were turned on until nose‐poke) and latency to consume the reward (timed from milkshake delivery until consumption) were recorded. Any nose‐poke occurring after a correct response but before reward consumption was considered a perseverative response (Figure S2B). In this task mice typically performed few, if any, premature responses (i.e., responses during the ITI) due to the short ITI length (2 s) and the relatively large size of the box.

#### 2.2.11 Home Cage Milkshake Consumption and Preference

Milkshake consumption and preference were investigated since alterations might impact operant task performance. Milkshake consumption and preference were measured over two non‐consecutive evenings (17:30–1:00) during which mice were single‐housed and exposed to strawberry milkshake ad libitum in a clean but familiar cage. Milkshake consumption was measured by presenting mice with a bottle containing milkshake (plus freely available food but no water bottle) which was weighed pre‐test and at 1 and 4 h of the test period. On a separate evening, mice underwent a milkshake preference test (7 h), which involved simultaneous presentation of two bottles, one containing milkshake and the other water (food available ad libitum). Bottles were switched locations after 3.5 h to avoid side preference. Milkshake preference was measured as milkshake minus water consumption.

#### 2.2.12 Spatial Novelty Preference Test

To measure short‐term spatial memory, a spatial novelty preference test was carried out as previously described [48, 55]. The apparatus consisted of a transparent Perspex 3‐arm Y‐maze (30 × 8 × 20 cm) lined with sawdust bedding which was placed in a dimly lit room containing a variety of extra‐maze cues. Before testing, each mouse was assigned a ‘familiar arm’ and a ‘novel arm’, counterbalanced between groups. At the beginning of the trial, the novel arm was closed off with a partition and the mouse was placed in the ‘start arm’ and allowed to explore this and the familiar arm for 5 min, before being placed back in its home cage. After 60 s, during which the partition was removed and the sawdust bedding was flattened, the mouse was placed back into the Y‐maze start arm and left free to explore all three arms for 2 min. Movements were recorded by an overhead camera and time spent in each arm was measured offline.

#### 2.2.13 Fear Conditioning

Fear conditioning was carried out as previously described [15] to assess aversive learning and memory. The experimental chamber (BIOSEB) comprised black methacrylate walls, a grid floor and a transparent ceiling and door. The conditioned stimulus (CS) was a tone (30 s, 2 s, 4000 Hz, 90 dB), while the unconditioned stimulus (US) consisted of a 0.25‐mA electric foot‐shock (2 s) and sensor measurements of freezing were obtained automatically using Panlab software (BIOSEB). Video recordings (Multimedia Video Record) were taken for offline manual scoring for validating the automatic counts.

On the conditioning day, mice were placed in the chamber for 2 min prior to the CS being delivered, while the US coincided with the final 2 s of the CS. After a 30 s ITI, a second CS‐US pairing was presented. Memory tests were carried out the following day and included a contextual memory test and a cued memory test, performed 2 h later. For the contextual test, mice were placed back in the conditioning context for 6 min, during which neither the CS nor the US was presented. Conversely, in the cue test mice spent 3 min exploring a novel context (characterised by different colour, shape, light and smell), prior to a continuous CS presentation for 3 min. Data were presented as percentage time spent freezing in 30 s time bins.

### 2.3 Data and Statistical Analysis

The Shapiro–Wilk test for normality was applied to all datasets. If data were normally distributed then a Student's *t*‐test, a two‐way ANOVA or a mixed‐effect model was carried out as appropriate. Specifically, a two‐way ANOVA was used on balanced datasets (i.e., same group size and no missing values), while a mixed effect model was employed in presence of unbalanced datasets. In the presence of statistically significant interactions, two‐way ANOVAs or mixed effect models were followed by Tukey's post hoc comparisons for balanced data, while Šídák's post hoc was selected for unbalanced data. If data were non‐parametric then a Mann–Whitney test was employed where appropriate. Parametric data are presented as mean ± standard error of the mean (SEM) values, whilst non‐parametric data are presented as median ± interquartile range values; *p* < 0.05 was considered statistically significant. GraphPad Prism (v10) was used for analysis and plotting of graphs.

An initial preliminary data analysis of both genotype and sex as between‐subject factors detected no effect of sex, nor sex × genotype interaction with the exception of data from the social preference test (see later). Thus, data were analysed only with genotype as a between‐subject factor.

All animals and datapoints were included in the analyses and graphs, as no outlier removal test was performed. However, some latency values from the appetitively motivated operant task were missing due to technical issues with the operant apparatus.

## 3 Results

### 3.1 Tests of Anxiety‐Like Behaviour and Locomotion

**FIGURE 2:**
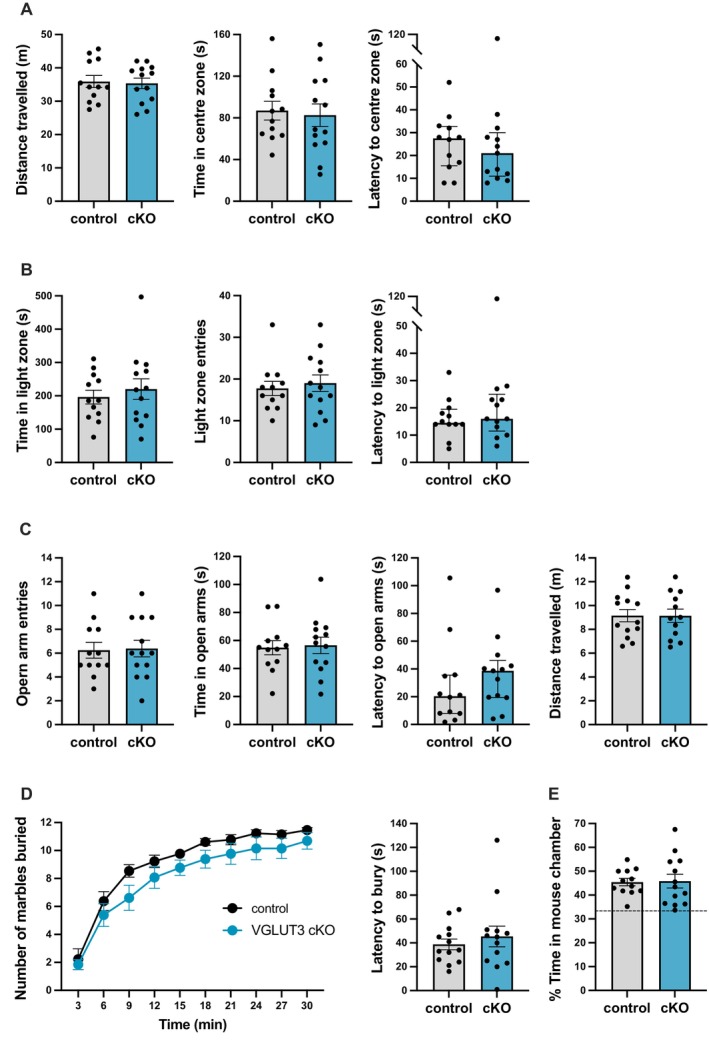
Performance of VGLUT3 cKO^5‐HT^ mice in tests of anxiety‐like behaviour compared to controls. (A) Open field, (B) light/dark box and (C) elevated plus maze in VGLUT3 cKO^5‐HT^ mice (*n* = 13) and littermate controls (*n* = 12). Bars represent the mean ± SEM values, except latencies which are median ± interquartile range. Individual values are indicated by closed circles. (D) Marble burying test in VGLUT3 cKO^5‐HT^ mice (*n* = 13) and controls (*n* = 13). Closed circles (left) and bars (right) represent mean ± SEM values. Individual values for latencies are indicated by smaller closed circles. (E) Performance in the social preference test in VGLUT3 cKO^5‐HT^ mice (*n* = 12) and controls (*n* = 13). Bars represent mean ± SEM values, with individual values indicated by closed circles. Chance is represented by the dashed line (33%). Data were analysed with unpaired *t*‐test, Mann–Whitney test (latencies) and two‐way repeated measures ANOVA (marble burying).

Previous evidence, obtained in mice with global VGLUT3 KO, suggested a role for VGLUT3 in anxiety‐related behaviours [8]. In the present study we used a mouse line lacking VGLUT3 specifically in 5‐HT neurons to further investigate the behavioural role of VGLUT3 in this neuronal population. First, to assess the potentially confounding effect of a locomotor phenotype on anxiety measures VGLUT3 cKO^5‐HT^ mice were tested using a non‐anxiogenic open field. Total distance travelled (*t*_(23)_ = 0.229, *p* = 0.821; Figure 2A), time spent in the centre zone (*t*_(23)_ = 0.305, *p* = 0.763; Figure 2A) and latency to enter (Mann–Whitney *U* = 65, *p* = 0.494; Figure 2A) the centre zone were not different between VGLUT3 cKO^5‐HT^ mice and control littermates. Time spent in the centre zone was 13.8% ± 1.8% and 14.5% ± 1.5% of total time in the open field for VGLUT3 cKO^5‐HT^ mice and control littermates, respectively.

To measure anxiety‐like behaviour, VGLUT3 cKO^5‐HT^ mice and littermate controls were tested in the light/dark box, EPM, marble burying test and social preference test. In the light/dark box, VGLUT3 cKO^5‐HT^ mice showed no difference in the time spent (*t*_(23)_ = 0.634, *p* = 0.533; Figure 2B), latency to enter (Mann–Whitney *U* = 63.5, *p* = 0.444; Figure 2B) or total entries (*t*_(23)_ = 0.477, *p* = 0.638; Figure 2B) into the light compartment, compared to controls. Time spent in the light zone was 34% ± 4.5% of total time in the apparatus for VGLUT3 cKO^5‐HT^ mice and 30.7% ± 3.9% for control littermates.

The EPM also did not reveal differences between VGLUT3 cKO^5‐HT^ mice and control littermates. There was no effect of genotype on the number of open arm entries (*t*_(23)_ = 0.139, *p* = 0.891; Figure 2C), time spent in the open arms (*t*_(23)_ = 2.213, *p* = 0.833; Figure 2C), latency to first enter the open arms (Mann–Whitney *U* = 56.5, *p* = 0.253; Figure 2C) or total distance travelled (*t*_(23)_ = 0.02, *p* = 0.984; Figure 2C). Time spent in open arms was 18.9% ± 2% of total time on the apparatus for VGLUT3 cKO^5‐HT^ mice and 16.9% ± 2.2% for control littermates.

The marble burying test revealed no statistically significant difference in the number of marbles buried over time by VGLUT3 cKO^5‐HT^ mice and control littermates (*F*_(1, 24)_ = 2.42, *p* = 0.133; Figure 2D) and there was no interaction between time and genotype (*F*_(9, 216)_ = 0.596, *p* = 0.800; Figure 2D). The latency to start burying was also not different between groups (*t*_(24)_ = 0.679, *p* = 0.504; Figure 2D).

In the social preference test both VGLUT3 cKO^5‐HT^ mice and control littermates spent significantly more time in the chamber with the unfamiliar mouse, compared to that with the novel object (control: *t*_(22)_ = 2.609; *p* = 0.016; VGLUT3 cKO^5‐HT^: *t*_(24)_ = 2.995, *p* = 0.006), but there was no difference between groups (*F*_(1, 21)_ = 0.164, *p* = 0.690; Figure 2E). A two‐way ANOVA found both an effect of sex (*F*_(1, 21)_ = 10.69, *p* = 0.004) and an interaction between sex and genotype (*F*_(1, 21)_ = 11.25, *p* = 0.003), as male VGLUT3 cKO^5‐HT^ mice spent more time in the chamber with the unfamiliar mouse (Tukey's post hoc: *p* = 0.041; Figure S3) compared to male controls. Conversely, female VGLUT3 cKO^5‐HT^ mice spent less time with the unfamiliar mouse (Tukey's post hoc: *p* = 0.0004; Figure S3) compared to male VGLUT3 cKO^5‐HT^ mice, but their performance was not different from female controls (Tukey's post hoc: *p* = 0.240; Figure S3).

There was no effect of sex or interaction between sex and genotype in any other behavioural tests presented here. Altogether, data presented in this section indicate no evidence of altered anxiety‐like behaviour in VGLUT3 cKO^5‐HT^ mice compared to control littermates.

### 3.2 Appetitively Motivated Behavioural Tests

Previous evidence implicated glutamate co‐released from 5‐HT neurons in reward processing [11, 23]. Thus, VGLUT3 cKO^5‐HT^ mice and littermate controls were assessed in appetitively motivated tests of anxiety (novelty‐induced hyponeophagia), anhedonia (sucrose preference test) and learning (appetitively motivated spatial reference memory test and operant paradigm).

**FIGURE 3:**
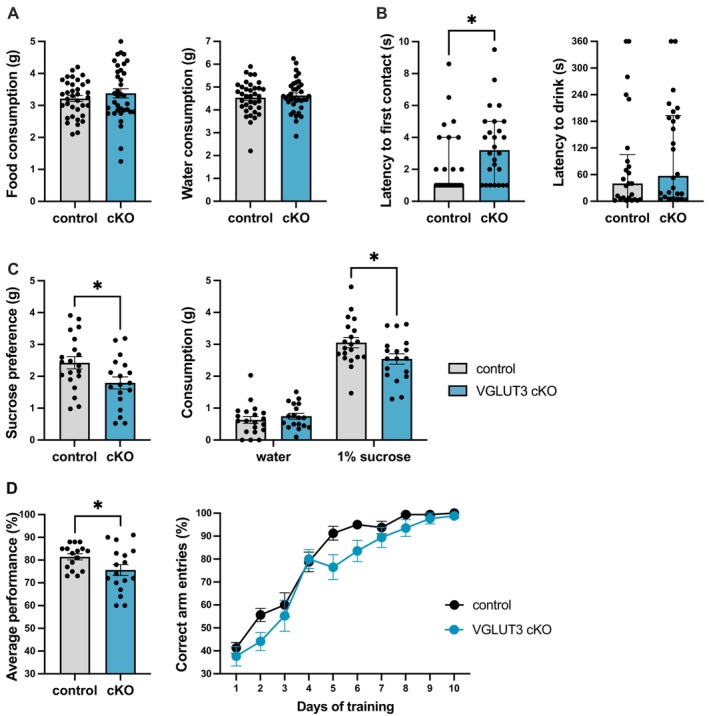
Performance of VGLUT3 cKO^5‐HT^ mice and controls in appetitively motivated tests. (A) Measurements of food and water consumption in VGLUT3 cKO^5‐HT^ mice (*n* = 36) and littermate controls (*n* = 36). Bars represent mean ± SEM values. Analysed by *t*‐test. (B) Novelty‐induced hyponeophagia in VGLUT3 cKO^5‐HT^ mice (*n* = 26) and controls (*n* = 25). Bars represent median ± interquartile range of latencies. Analysed by Mann–Whitney *U*. (C) Sucrose preference score and raw values for water and 1% sucrose consumption in VGLUT3 cKO^5‐HT^ mice (*n* = 18) and controls (*n* = 20) during the sucrose preference test. Bars represent mean ± SEM values. Analysed by two‐way ANOVA followed by Šídák's post hoc. (D) Average performance across training days and percentage of correct trials per day performed by VGLUT3 cKO^5‐HT^ mice (*n* = 17) and controls (*n* = 16) in the appetitively motivated spatial reference Y maze. Full circles represent mean ± SEM values, chance = 33%. Some error bars are too small to be depicted. Analysed by two‐way repeated measure ANOVA. *p < 0.05.

First, home cage food and water consumption were assessed in VGLUT3 cKO^5‐HT^ mice to ensure that they did not differ from controls and therefore unlikely to influence appetitively motivated behavioural measurements. VGLUT3 cKO^5‐HT^ mice did not show differences in their home cage consumption of food (*t*_(70)_ = 0.966, *p* = 0.337; Figure 3A) or water (*t*_(70)_ = 0.551, *p* = 0.584; Figure 3A) compared to control littermates. Similarly, average body weight was not different between genotypes (data not shown).

#### 3.2.1 Novelty‐Induced Hyponeophagia

In the novelty‐induced hyponeophagia test VGLUT3 cKO^5‐HT^ mice showed a modest increase in latency to first contact with the novel food compared to control littermates (Mann–Whitney *U* = 216, *p* = 0.033; Figure 3B). However, latency to first drink was not different between groups (Mann–Whitney *U* = 275, *p* = 0.351; Figure 3B). The latter readout is typically used as a measure of anxiety in this test [47]. Therefore, these data together suggest no difference in anxiety‐like behaviour between groups. VGLUT3 cKO^5‐HT^ mice did not differ from controls in terms of weight loss following food restriction (data not shown).

#### 3.2.2 Sucrose Preference Test

In the sucrose preference test, both VGLUT3 cKO^5‐HT^ mice and controls consumed significantly more 1% sucrose solution than water (*F*_(1, 36)_ = 243.9, *p* < 0.0001; Figure 3C). Interestingly, a two‐way ANOVA revealed an interaction between genotype and sucrose solution (*F*_(1, 36)_ = 5.455, *p* = 0.025), with Šídák's post hoc indicating that VGLUT3 cKO^5‐HT^ mice drank less sucrose than controls (*p* = 0.019; Figure 3C), while water consumption was unchanged (*p* = 0.795). Therefore, the sucrose preference score was reduced in VGLUT3 cKO^5‐HT^ mice compared to controls (*p* = 0.025; Figure 3C), suggesting that VGLUT3 cKO^5‐HT^ mice showed a decreased preference for sucrose over water.

Prior exposure of VGLUT3 cKO^5‐HT^ mice to a single bottle of 2.5% sucrose solution to avoid neophobia revealed no difference in consumption between these mice and controls (1.7 ± 0.2 g vs. 1.9 ± 0.2 g, respectively; *t*_(36)_ = 0.599, *p* = 0.530).

#### 3.2.3 Appetitively Motivated Spatial Reference Memory Test

In the appetitively motivated Y‐maze task, all mice effectively learned the task over time (effect of training day: *F*_(9, 279)_ = 87.020, *p* < 0.0001; Figure 3D). However, VGLUT3 cKO^5‐HT^ mice showed a reduction in overall performance compared to control littermates (effect of genotype: *F*_(1, 31)_ = 4.246, *p* = 0.048; Figure 3D). The interaction between genotype and day was not statistically significant (*F*_(9, 279)_ = 1.233, *p* = 0.275). In the last training session (Day 10), post‐choice baiting confirmed that mice were not relying on olfactory cues. Overall, this result suggests a reduction in appetitively motivated learning in VGLUT3 cKO^5‐HT^ mice compared to control littermates.

#### 3.2.4 Appetitively Motivated Operant Paradigm

**FIGURE 4:**
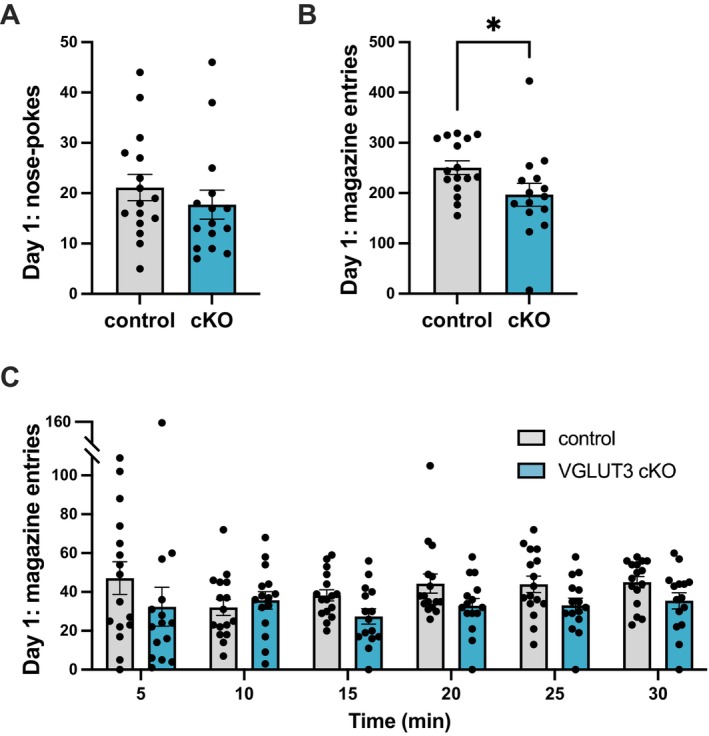
Performance of VGLUT3 cKO^5‐HT^ mice and controls in the habituation session (Day 1) of the appetitively motivated operant paradigm. (A) Nose‐pokes in the five ports, which were not illuminated at this time. (B) Number of magazine entries during the whole 30 min session. (C) Number of magazine entries during the whole session in 5 min time bins. Bars represent mean ± SEM values, with individual values indicated by closed circles. Data from VGLUT3 cKO^5‐HT^ mice (*n* = 15) and control littermates (*n* = 17) were analysed by *t*‐test or two‐way repeated measures ANOVA. *p < 0.05.

To further investigate learning with reward, food‐restricted VGLUT3 cKO^5‐HT^ mice and control littermates were tested in an appetitively motivated operant paradigm. During the first day of exposure to the operant boxes, in which mice received five milkshake deliveries (without needing to nose‐poke), VGLUT3 cKO^5‐HT^ mice had a reduced number of magazine entries compared to littermate controls (*F*_(1, 29)_ = 4.254, *p* = 0.048; Figure 4B). Analysis of magazine entries in 5 min time bins found no significant effect of time bin (*F*_(5, 145)_ = 0.932, *p* = 0.462; Figure 4C), nor interaction between time bin and genotype (*F*_(5, 145)_ = 0.962, *p* = 0.443), suggesting that the reduction in magazine entries was uniform across the whole session. The total number of nose‐pokes in the five ports (which were not illuminated at this stage) was unchanged between groups (*t*_(29)_ = 0.875, *p* = 0.389; Figure 4A), suggesting similar levels of exploration of the operant chamber.

**FIGURE 5:**
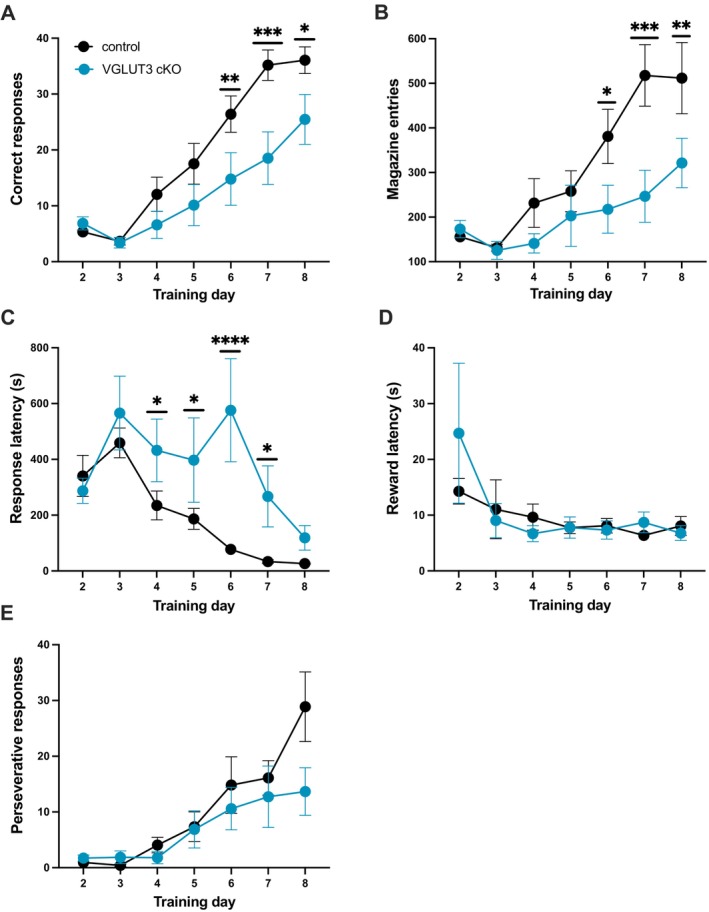
Performance of VGLUT3 cKO^5‐HT^ mice and controls in the appetitively motivated operant paradigm. (A) Number of correct responses, (B) number of magazine entries, (C) response latency, (D) reward latency, (E) number of perseverative responses. Closed circles represent mean ± SEM values (some error bars are too small to be depicted) for VGLUT3 cKO^5‐HT^ mice (*n* = 15) and control littermates (*n* = 17). Data were analysed by repeated measures mixed‐effect model, followed by Tukey's test. ****p < 0.0001, ***p < 0.001, **p < 0.01, *p < 0.05.

In the following 2 days of training in the appetitively motivated operant paradigm (Days 2–3; Figure 5A,B), the two groups of mice were well‐matched for performance in terms of correct responses and magazine entries. Subsequently (Days 4–8; Figure 5A), all mice displayed an increased number of correct responses over the 7 days of training (effect of training day: *F*_(6, 180)_ = 50.65, *p* < 0.0001; Figure 5A). However, VGLUT3 cKO^5‐HT^ mice displayed a reduced number of correct responses compared to control littermates (effect of genotype: *F*_(1, 30)_ = 4.723, *p* = 0.038; genotype × training day interaction: *F*_(6, 180)_ = 4.752, *p* = 0.0002; Figure 5A). Tukey's post hoc comparisons revealed that this effect was significant on training Days 6, 7 and 8 (*p* = 0.008, 0.0001 and 0.014 respectively). VGLUT3 cKO^5‐HT^ mice also demonstrated a decreased number of entries in the magazine compared to controls (effect of genotype: *F*_(1, 30)_ = 5.790, *p* = 0.023; genotype × training day interaction: *F*_(6, 180)_ = 2.98, *p* = 0.009; Figure 5B), again with a significant difference on Days 6, 7 and 8 (*p* = 0.024, 0.0002 and 0.0009 respectively). Analysis of the number of perseverative responses revealed no effect of genotype, nor genotype × day interaction (*F*_(1, 30)_ = 1.390, *p* = 0.248, *F*_(6, 180)_ = 1.800, *p* = 0.102; Figure 5E).

Additionally, VGLUT3 cKO^5‐HT^ mice were slower to nose‐poke the light stimuli compared to control littermates (response latency: effect of genotype: *F*_(1, 30)_ = 9.61, *p* = 0.004; genotype × day interaction: *F*_(6, 158)_ = 3.28, *p* = 0.005; Figure 5C), with a significant difference on Days 4, 5, 6 and 7 (*p* = 0.049, 0.025, < 0.0001, 0.047). In contrast, there was no difference between groups in the latency to consume the milkshake reward (reward latency: effect of genotype: *F*_(1, 30)_ = 0.201, *p* = 0.657; genotype × day interaction: *F*_(6, 160)_ = 0.690, *p* = 0.658; Figure 5D).

**FIGURE 6:**
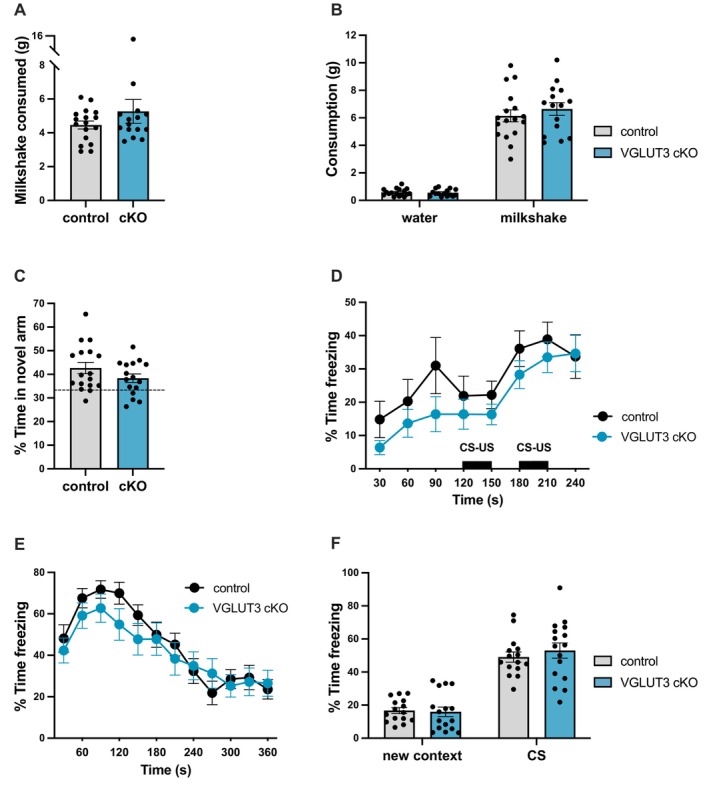
Tests probing appetite, short‐term memory and aversive associative learning in VGLUT3 cKO^5‐HT^ mice and controls. (A) Measurements of milkshake consumption (one bottle test) in VGLUT3 cKO^5‐HT^ mice (*n* = 15) and control littermates (*n* = 17). (B) Raw values for water and milkshake consumption by VGLUT3 cKO^5‐HT^ mice (*n* = 15) and control littermates (*n* = 17) in the milkshake preference test. (C) Percentage time spent in the novel arm of the spatial novelty preference test in VGLUT3 cKO^5‐HT^ mice (*n* = 17) and control littermates (*n* = 16). (D–F) Percentage time spent freezing during fear conditioning, contextual and cued test in VGLUT3 cKO^5‐HT^ mice (*n* = 16) and controls (*n* = 15). (A–C, F) Bars are mean ± SEM and individual values are indicated by closed circles, while in (D, E) closed circles connected with lines represent mean ± SEM. Data were analysed by *t*‐test or two‐way ANOVA.

A possible explanation for VGLUT3 cKO^5‐HT^ mice showing a performance deficit in the appetitively motivated operant task could be reduced sensitivity for the milkshake reward. To rule out this possibility, VGLUT3 cKO^5‐HT^ mice were exposed to milkshake in a one‐bottle test. Compared to littermate controls, VGLUT3 cKO^5‐HT^ mice showed no difference in consumption at 1 h (*t*_(30)_ = 1.132, *p* = 0.267; Figure 6A) or 4 h (*t*_(30)_ = 0.619, *p* = 0.541; Figure S4A). Similarly, when presented with the choice of either milkshake or water, both genotypes consumed significantly more milkshake (main effect of solution: *F*_(1, 30)_ = 321.200, *p* < 0.0001) but there was no effect of genotype (*F*_(1, 30)_ = 0.544, *p* = 0.467; Figure 6B), nor interaction between genotype and solution (*F*_(1, 30)_ = 0.652, *p* = 0.423).

Overall, VGLUT3 cKO^5‐HT^ mice displayed reduced performance in this appetitively motivated operant paradigm compared to controls, without altered milkshake preference, suggesting impaired learning for reward.

### 3.3 Learning in Spatial Novelty Preference and Fear Memory Tests

As VGLUT3 cKO^5‐HT^ mice showed reduced performance in appetitively motivated learning tests compared to controls, to understand the specificity of these deficits, further tests probed learning without rewards, specifically short‐term spatial memory (spatial novelty preference test) and aversive associative learning (fear conditioning).

#### 3.3.1 Spatial Novelty Preference Test

The spatial novelty preference test was used to assess spatial short‐term memory in VGLUT3 cKO^5‐HT^ mice and control littermates in the absence of any US. Both groups showed a preference for the novel arm (performance of controls versus chance: *t*_(16)_ = 3.923, *p* = 0.001; performance of VGLUT3 cKO^5‐HT^ mice versus chance: *t*_(16)_ = 2.789, *p* = 0.014; Figure 6C) and there was no statistically significant difference in percentage time spent in the novel arm (*t*_(31)_ = 1.430, *p* = 0.163; Figure 6C) between VGLUT3 cKO^5‐HT^ mice and controls. Raw data of time spent in each arm is depicted in Figure S4B.

#### 3.3.2 Fear Conditioning

A fear conditioning test was also used to assess fear learning and memory in VGLUT3 cKO^5‐HT^ mice and control littermates. During training, which involved presentation of two CS‐US pairings, % of time spent freezing did not differ between groups (*F*_(1, 29)_ = 1.677, *p* = 0.206; Figure 6D). As expected, both groups showed an increase in freezing over the course of the training (effect of time: *F*_(7, 203)_ = 10.62, *p* < 0.0001), but there was no difference between genotypes (time × genotype interaction: *F*_(7, 203)_ = 0.572, *p* = 0.778).

Similarly in a context fear memory test, during which mice were returned to the fear conditioning context, VGLUT3 cKO^5‐HT^ mice spent the same proportion of time freezing (*F*_(1, 29)_ = 0.362, *p* = 0.552; Figure 6E) as controls. Both groups displayed reduced freezing over the course of this session (effect of time: *F*_(11, 319)_ = 28.55, *p* < 0.001), but there was no difference between genotypes across time (time × genotype interaction: *F*_(11, 319)_ = 1.366; *p* = 0.188).

Finally, in a cued‐fear memory test, involving the presentation of the CS in a new context, both groups displayed enhanced freezing during CS exposure (effect of time: *F*_(1, 29)_ = 227.1, *p* < 0.0001), but there was also no effect of genotype (*F*_(1, 29)_ = 0.148, *p* = 0.703) and no interaction between time and genotype (*F*_(1, 29)_ = 1.00, *p* = 0.325; Figure 6F; see Figure S4C for data presented in 30 s time bins).

### 3.4 Phenotype of SERT‐Cre Mice

As VGLUT3 cKO^5‐HT^ mice express a transgene that drives Cre recombinase under the control of the SERT promoter, experiments also compared SERT‐Cre mice with wildtype controls to ensure that this genotype alone is not sufficient to produce phenotypic changes.

In the appetitively motivated operant task, SERT‐Cre mice showed a non‐significant *increase* in correct responses compared to controls (*F*_(1, 14)_ = 3.799, *p* = 0.072; Figure S5C), unlike VGLUT3 cKO^5‐HT^ mice which displayed a *reduction* in correct responses. SERT‐Cre mice did not differ from wildtypes in any other parameters (see Figure SFA–F), including number of magazine head entries (effect of genotype: *F*_(1, 14)_ = 1.118, *p* = 0.308; genotype × time interaction: *F*_(6, 82)_ = 0.650, *p* = 0.690; Figure S5D) or latency to nose poke into the illuminated ports (effect of genotype: *F*_(1, 14)_ = 1.855, *p* = 0.195; genotype × time interaction: *F*_(6, 82)_ = 0.372, *p* = 0.895; Figure S5E). These data suggest that Cre expression per se was not responsible for the reduction in performance of VGLUT3 cKO^5‐HT^ mice in the appetitively motivated operant task.

SERT‐Cre mice were also assessed in paradigms of anxiety and anhedonia, including the EPM, novelty‐induced hyponeophagia and sucrose preference test. In the EPM their behaviour did not differ from that of littermate controls in the number of open arm entries (*t*_(17)_ = 0.335, *p* = 0.742; Table S2), time spent in the open arms (*t*_(17)_ = 0.819, *p* = 0.424) and total distance travelled (*t*_(17)_ = 0.575, *p* = 0.573). In the novelty‐induced hyponeophagia test in which VGLUT3 cKO^5‐HT^ mice showed reduced latency to first contact, performance of SERT‐Cre mice was not different from controls in terms of either latency to first contact (Mann–Whitney *U* = 50, *p* = 0.364) or latency to drink (*t*_(21)_ = 0.214, *p* = 0.833; Table S2). Finally, unlike VGLUT3 cKO^5‐HT^ mice, SERT‐Cre mice showed no evidence of reduced sucrose preference compared to controls (*t*_(13)_ = 1.062, *p* = 0.308; Table S2). Altogether, SERT‐Cre mice showed no behavioural phenotype alteration in the tests performed here.

## 4 Discussion

Here, we used VGLUT3 cKO^5‐HT^ mice to investigate the role of VGLUT3 in 5‐HT neurons in a variety of behaviours and cognitions, including anxiety‐like behaviour, appetitively motivated behaviour, as well as learning in other contexts. Contrary to predictions from previous studies using mice with a global VGLUT3 KO [8], VGLUT3 cKO^5‐HT^ mice did not show evidence of increased anxiety‐like behaviour. However, potentially in keeping with other earlier evidence of a role for co‐released glutamate in reward processing [11, 23], VGLUT3 cKO^5‐HT^ mice displayed reduced performance in tasks involving learning with reward, as well as modest deficits in other tasks with an element of appetitive motivation. Behaviour of VGLUT3 cKO^5‐HT^ mice was not different from controls in tests of Pavlovian cue and context fear conditioning, nor during a novelty preference test of spatial short‐term memory. Taken together, these data are consistent with VGLUT3 in 5‐HT neurons being involved in some aspects of learning, here in association with learning for reward.

VGLUT3 cKO^5‐HT^ mice demonstrate a loss of VGLUT3 in midbrain 5‐HT neurons [30, 31] but, unlike their control littermates, express a transgene that drives Cre recombinase under the control of the SERT promoter. Importantly, however, mice expressing the SERT‐Cre transgene alone did not differ from wildtype controls in tasks such as the appetitively motivated operant task in which VGLUT3 cKO^5‐HT^ mice demonstrated a deficit. This suggests that Cre expression alone does not contribute to the behavioural phenotype of VGLUT3 cKO^5‐HT^ mice observed here.

### 4.1 VGLUT3 cKO^5^ ^‐HT^ Mice Did Not Display an Anxious Phenotype

VGLUT3 cKO^5‐HT^ mice showed no difference from littermate controls in the EPM and light/dark box, two well‐established tests assessing anxiety‐like behaviour [56]. Additionally, VGLUT3 cKO^5‐HT^ mice did not differ from controls in the time spent in the centre zone of the non‐anxiogenic open field. In these tests, mice spent the expected amount of time in the more anxiogenic area of the apparatus; thus, the lack of differences between groups is not likely caused by floor or ceiling effects. Distance travelled in the non‐anxiogenic open field, EPM and light/dark box was also not different between VGLUT3 cKO^5‐HT^ mice and controls, suggesting similar levels of locomotion.

VGLUT3 cKO^5‐HT^ mice and control littermates were also exposed to a social interaction test, as reduced sociability can indicate anxiety‐like behaviour [45]. However, both groups showed similar levels of social interaction with a novel conspecific. VGLUT3 cKO^5‐HT^ mice also performed similarly to controls in the marble burying test, which has been previously used to assess neophobia and repetitive behaviour [43, 57, 58]. Taken together, these data suggest that VGLUT3 cKO^5‐HT^ mice do not have an anxious phenotype.

However, in the novelty‐induced hyponeophagia test mice were slower to approach a novel sweet solution (sweetened condensed milk) in an unfamiliar environment, which is considered a readout of anxiety‐like behaviour [58]. Nonetheless, it is important to note that the majority of mice had a latency of 3 s or less to contact the food. Additionally, VGLUT3 cKO^5‐HT^ mice did not differ from controls in the latency to consume the novel food, which is the main anxiety readout of the novelty‐induced hyponeophagia test. Therefore, these data together suggest no difference in anxiety‐like behaviour between groups.

This evidence that VGLUT3 cKO^5‐HT^ mice have normal levels of anxiety and locomotion, contrasts with a report of hypolocomotion in mice with selective VGLUT3 deletion in neurons expressing the 5‐HT regulator gene Pet1 [22]. Specifically, the latter mice showed reduced locomotion in the open field and EPM, as well as increased preference for the dark compartment of the light/dark box [22]. However, the phenotype of the Pet1 mutant mice is complex to interpret because of the occurrence of Pet1 expression in midbrain raphe neurons which lack detectable levels of 5‐HT‐related genes [59, 60].

VGLUT3 cKO^5‐HT^ mice also differ from mice with global VGLUT3 KO, which exhibited increased anxiety‐like behaviour in the EPM, novelty‐induced hyponeophagia and marble burying tests [8]. Since VGLUT3 is also expressed in populations of GABAergic, cholinergic and glutamatergic neurons [5, 6, 7, 18], it is likely that the anxiety phenotype observed in global VGLUT3 KO mice results from the loss of VGLUT3 in these other neuronal populations rather than in 5‐HT neurons alone.

### 4.2 VGLUT3 cKO^5^ ^‐HT^ Mice Showed Deficits in Appetitively Motivated Tasks

Interestingly, VGLUT3 cKO^5‐HT^ mice showed a modest reduction in preference for a 1% sucrose solution, compared to controls. Although in a separate test VGLUT3 cKO^5‐HT^ mice did not show reduced preference for milkshake, the latter contains a higher concentration of sucrose (8.7%). Indeed, there is consistent evidence that preference sensitivity is lost in tests using sucrose‐rich solutions (> 2%) [50, 52, 61, 62, 63].

A reduction in sucrose preference is often interpreted as anhedonia, a core feature of a depressive‐like phenotype [64]. However, results from the sucrose preference test are difficult to interpret, as this paradigm may be sensitive to both hedonic (‘liking’) and motivational (‘wanting’) aspects of the reward. Additionally, based on previous in vivo optogenetic evidence linking 5‐HT‐glutamate co‐release to reward processing [11, 23], the reduced sucrose preference observed in VGLUT3 cKO^5‐HT^ mice may be better explained as diminished reward sensitivity rather than simply evidence of an anhedonic‐like phenotype.

Following these data, the phenotype of VGLUT3 cKO^5‐HT^ mice was further investigated using paradigms involving learning with reward. When exposed to an appetitively motivated Y maze task in which mice use extra‐maze spatial cues to learn the location of a reward, VGLUT3 cKO^5‐HT^ mice demonstrated a clear impairment in performance. Since this paradigm is essentially a multi‐trial place preference test, this result is in keeping with previous optogenetic evidence that 5‐HT and glutamate release are involved in the regulation of place preference [11, 23].

To further assess learning in the context of reward, VGLUT3 cKO^5‐HT^ mice were tested in an appetitively motivated operant task in which a nose‐poke at any of five illuminated ports triggered the delivery of a milkshake reward from a separate magazine. During training, VGLUT3 cKO^5‐HT^ mice demonstrated a reduced number of responses to the illuminated ports, fewer magazine entries and were slower to respond to the light cue compared to control littermates. On all these parameters, VGLUT3 cKO^5‐HT^ mice were able to improve over time but did so at a slower rate compared to controls.

This finding that VGLUT3 cKO^5‐HT^ mice had a deficit in the operant task suggests that VGLUT3 in 5‐HT neurons is critical for appetitively motivated learning. There are, however, a number of possible explanations for the poor performance in this task including reduced interest in the reward. The latter idea is consistent with the finding that on the first day of habituation training (when mice received five rewards without having to nose‐poke), VGLUT3 cKO^5‐HT^ mice approached the magazine less frequently than controls. However, when VGLUT3 cKO^5‐HT^ mice were presented with the same appetitive reward (milkshake) in their home cage neither their consumption nor preference for milkshake were different from controls. These findings suggest that the behavioural differences observed in the operant task are unlikely to result from reduced appetite for the milkshake reward. Moreover, performance of the two groups was well matched on the initial days of operant training.

Altogether, VGLUT3 cKO^5‐HT^ mice showed evidence of impairments in two appetitively motivated learning tasks, with no concurrent change in preference for the milkshake reward. Alternative explanations for the deficits in these tasks include specific Pavlovian or instrumental impairments or reduced motivation to work for rewards. To obtain further insights, VGLUT3 cKO^5‐HT^ mice were tested in additional learning paradigms.

### 4.3 VGLUT3 cKO^5^ ^‐HT^ Mice Were Unimpaired in Short‐Term Memory and Fear Memory Tasks

Performance of VGLUT3 cKO^5‐HT^ mice did not differ from controls in the spatial novelty preference test, suggesting unimpaired short‐term spatial memory. Additionally, relative to controls VGLUT3 cKO^5‐HT^ mice showed unaltered contextual and cued fear memory. This finding contrasts with previous reports indicating that global VGLUT3 KO mice exhibited learning deficits, including impaired spatial memory [16], as well as increased contextual fear and fear generalisation [14, 15]. However, as noted above regarding the increased anxiety of global VGLUT3 KO mice, this difference is likely explained by the loss of VGLUT3 in non‐5‐HT neurons.

As VGLUT3 cKO^5‐HT^ mice were impaired in appetitively motivated learning tasks, but showed no deficits in cued and contextual fear learning, it is tempting to conclude that VGLUT3 in 5‐HT neurons plays a specific role in reward‐based learning. However, fear conditioning involves forming simple Pavlovian associations between the conditioned stimuli (i.e., both the tone and the experimental context) and the aversive US. Conversely, the appetitively motivated operant task involved two distinct instrumental responses (nose‐poking of light cues and engaging with the drinking spout) performed at different spatial locations. Moreover, these responses must be generated in the correct sequence and at the appropriate time in relation to delivery of punctate Pavlovian stimuli (i.e., the light cues in the nose‐poke ports). Thus, there are a number of possible mechanisms through which VGLUT3 cKO^5‐HT^ mice might be impaired in the operant task, beyond simply a difference in the nature of the unconditioned stimuli (i.e., appetitive reward versus aversive stimulus), as discussed below.

### 4.4 A Role for VGLUT3 in 5‐HT Neurons in Learning for Reward

Overall, VGLUT3 cKO^5‐HT^ mice showed evidence of impairments in appetitively motivated tasks, with no concurrent changes in anxiety‐like behaviour or appetite. Although we found no evidence of deficits in the other learning paradigms used here, it remains possible that impairments in specific learning processes contribute to the observed phenotype of VGLUT3 cKO^5‐HT^ mice. For example, these mice may have deficits in instrumental learning (i.e., difficulties in learning the association between nose‐poking and reward delivery), Pavlovian learning (i.e., regarding the association between the light stimulus and reward availability) or acquisition of occasion setting [65].

Nevertheless, the finding that VGLUT3 cKO^5‐HT^ mice showed impairments in an appetitively motivated learning task is in line with previous evidence showing that optogenetic stimulation of Pet‐1 expressing DRN terminals projecting to the VTA is rewarding [11]. Furthermore, previous in vitro electrophysiological evidence indicates that VGLUT3 and SERT‐expressing neurons from the DRN establish asymmetric synapses onto VTA dopamine neurons and stimulate dopamine release in the nucleus accumbens, a key area for reward processing [23].

Thus, it is possible that the behavioural deficits displayed by VGLUT3 cKO^5‐HT^ mice might be caused by altered function of this mesolimbic dopamine pathway, due to changes in 5‐HT‐glutamate co‐signalling impacting on dopamine neurons via ionotropic or metabotropic glutamate receptor activation. Future concurrent recordings of dopamine neuronal activity/release during appetitively motivated tasks would inform on this possibility. However, it has also been suggested that VGLUT3 may promote vesicular loading of 5‐HT [8]. According to this hypothesis, provided that 5‐HT and glutamate are released from the same vesicle [1, 8] (but see also [12]), lack of VGLUT3 in 5‐HT neurons may result in a reduction of vesicular 5‐HT loading, which could contribute to the behavioural phenotype described here.

Finally, altered 5‐HT‐glutamate co‐signalling in other DRN projections might contribute to the observed learning phenotype. For example, DRN 5‐HT projections to the prefrontal cortex are both activated by reward [66] and involved in waiting for future rewards [67]. Furthermore, DRN 5‐HT neurons also project directly to the nucleus accumbens, which may also influence learning for reward [68, 69, 70].

## 5 Conclusions

Understanding the behavioural role of VGLUT3 co‐expressed in 5‐HT neurons is important in the context of recent evidence that VGLUT3 expression likely varies across the human population. This may be due to genetic factors such as mutations of the VGLUT3 gene (Slc17a8) [27, 71] or environmental factors such as exposure to stress or acquisition of generalised fear [28, 29]. The current data suggest that in such cases, a natural variation in VGLUT3 might affect a selective subset of learning mechanisms, here in association with learning for reward, resulting in altered behaviour.

## Funding

This work was supported by the Medical Research Council (MR/N013468/1), Oxford Medical Sciences Internal Fund: Pump‐Priming by the Nuffield Benefaction for Medicine, the Wellcome Institutional Strategic Support Fund (ISSF) (0005667) and French Ministry of Research.

## Ethics Statement

Experiments were carried out following local ethical review and in accordance with UK Animals (Scientific Procedures) Act of 1986, European Communities Council Directive (86/809/EEC) and the Ministère de l'Agriculture et de la Forêt, Service Vétérinaire de la Santé et de la Protection Animale.

## Conflicts of Interest

The authors declare no conflicts of interest.

## Supporting information


**Figure S1:** Breeding diagrams detailing the development of VGLUT3 cKO^5‐HT^ mice and current breeding strategy.
**Table S1:** Summary of the parameters employed in the appetitively motivated operant paradigm.
**Figure S2:** Photograph of the operant chamber setup together with illustration of the task used.
**Figure S3:** Additional data from the social preference test in VGLUT3 cKO^5‐HT^ mice and controls.
**Figure S4:** Additional data on milkshake consumption, spatial novelty preference and cued fear conditioning in VGLUT3 cKO^5‐HT^ mice and controls.
**Figure S5:** Performance of SERT‐Cre mice and controls in the appetitively motivated operant paradigm.
**Table S2:** Behaviour of SERT‐Cre mice compared to control littermates.

## Data Availability

The data that support the findings of this study are available from the corresponding author upon reasonable request.
