# Supplementary material for: Phenotypic Behavioural Effects of Genetic Deletion of the Vesicular Glutamate Transporter 3 in 5‐Hydroxytryptamine Neurons
Source: Genes Brain Behav. 2026 Jul 27;25(4):e70063. doi: 10.1111/gbb.70063 (PMC13402935; doi:10.1111/gbb.70063)
Supplement: Supplementary file 1 — Figure S1: Breeding diagrams detailing the development of VGLUT3 cKO5‐HT mice and current breeding strategy. Table S1: Summary of the parameters employed in the appetitively motivated operant paradigm. Figure S2: Photograph of the operant chamber setup together with illustration of the task used. Figure S3: Additional data from the social preference test in VGLUT3 cKO5‐HT mice and controls. Figure S4: Additional data on milkshake consumption, spatial novelty preference and cued fear conditioning in VGLUT3 cKO5‐HT mice and controls. Figure S5: Performance of SERT‐Cre mice and controls in the appetitively motivated operant paradigm. Table S2: Behaviour of SERT‐Cre mice compared to control littermates. [file GBB-25-e70063-s001.docx]

Supporting information

Phenotypic behavioural effects of genetic deletion of the vesicular glutamate transporter 3 in 5-hydroxytryptamine neurons

L. Sophie Gullino^1†*^, Nida Chabbah^2^, Spatika Jayaram^1^, Raquel Pinacho^3^, Salah El Mestikawy^2,4^, Stéphanie Daumas^2^, David M. Bannerman^3^, Trevor Sharp^1*^

^1^Department of Pharmacology, University of Oxford, Mansfield Road, Oxford OX1 3QT, UK; ^2^Sorbonne Université, INSERM, CNRS, Centre for Neuroscience (NeuroSU)– Institut de Biologie Paris Seine (IBPS), F-75005 Paris, France; ^3^Department of Experimental Psychology, University of Oxford, Mansfield Road, Oxford, OX1 3TA, UK; ^4^Douglas Mental Health University Institute, Department of Psychiatry, McGill University, Montreal, QC, H4H 1R3, Canada.

†Present address: Laboratory of Pharmaceutical Chemistry, Drug Analysis and Drug Information (FASC), Vrije Universiteit Brussel, 1090 Jette, Belgium.

*Correspondence: L. Sophie Gullino and Trevor Sharp


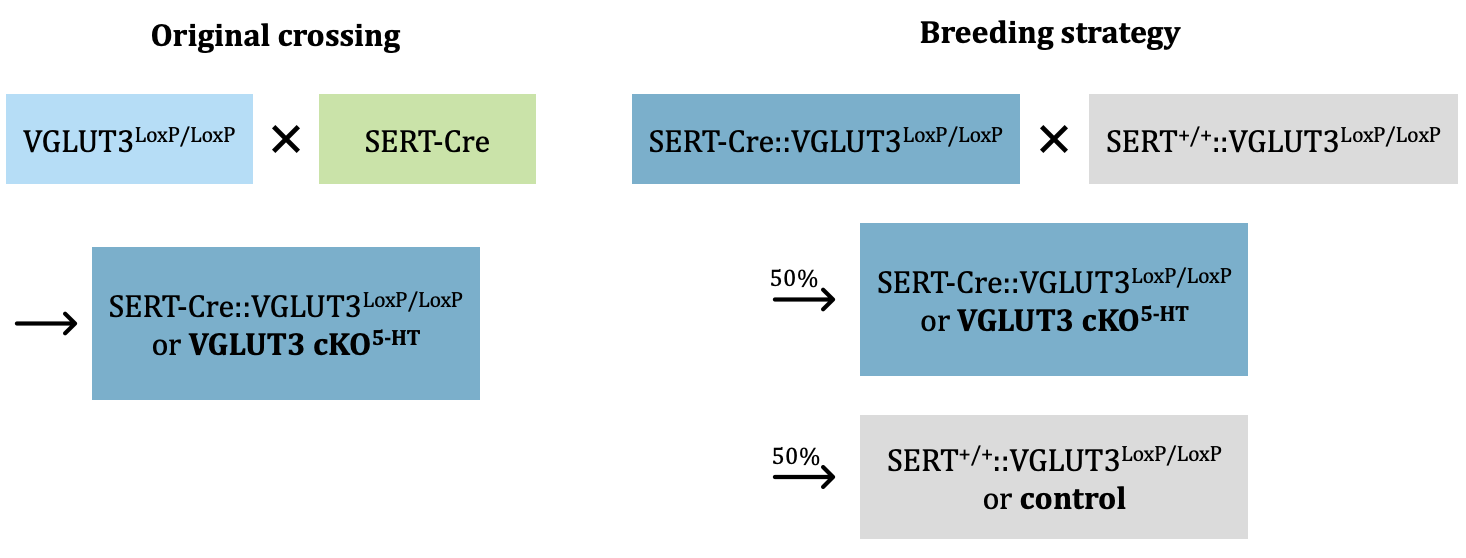


**Supplementary Figure 1 | Breeding diagrams detailing the development of VGLUT3 cKO^5‑HT^ mice and current breeding strategy.**

**Supplementary Table 1 | Summary of the parameters employed in the appetitively-motivated operant paradigm.**

| **Day and session** | **Task** | **Description** | **Stimulus duration** | **ITI** | **Session duration** |
| --- | --- | --- | --- | --- | --- |
| Day 1 | **Habituation** to the setup | No ports are illuminated. Delivery of 5 milkshake rewards (12 μl each) from the magazine. | NA | 5 min | 30 min |
| Day 2-8 | **Main task** | All 5 ports are illuminated, nose-poke of any illuminated port results in the port lights being switched off and triggers reward delivery (12 μl) from the magazine. | Until nose-poke | 2 s | 30 min or 40 correct responses |


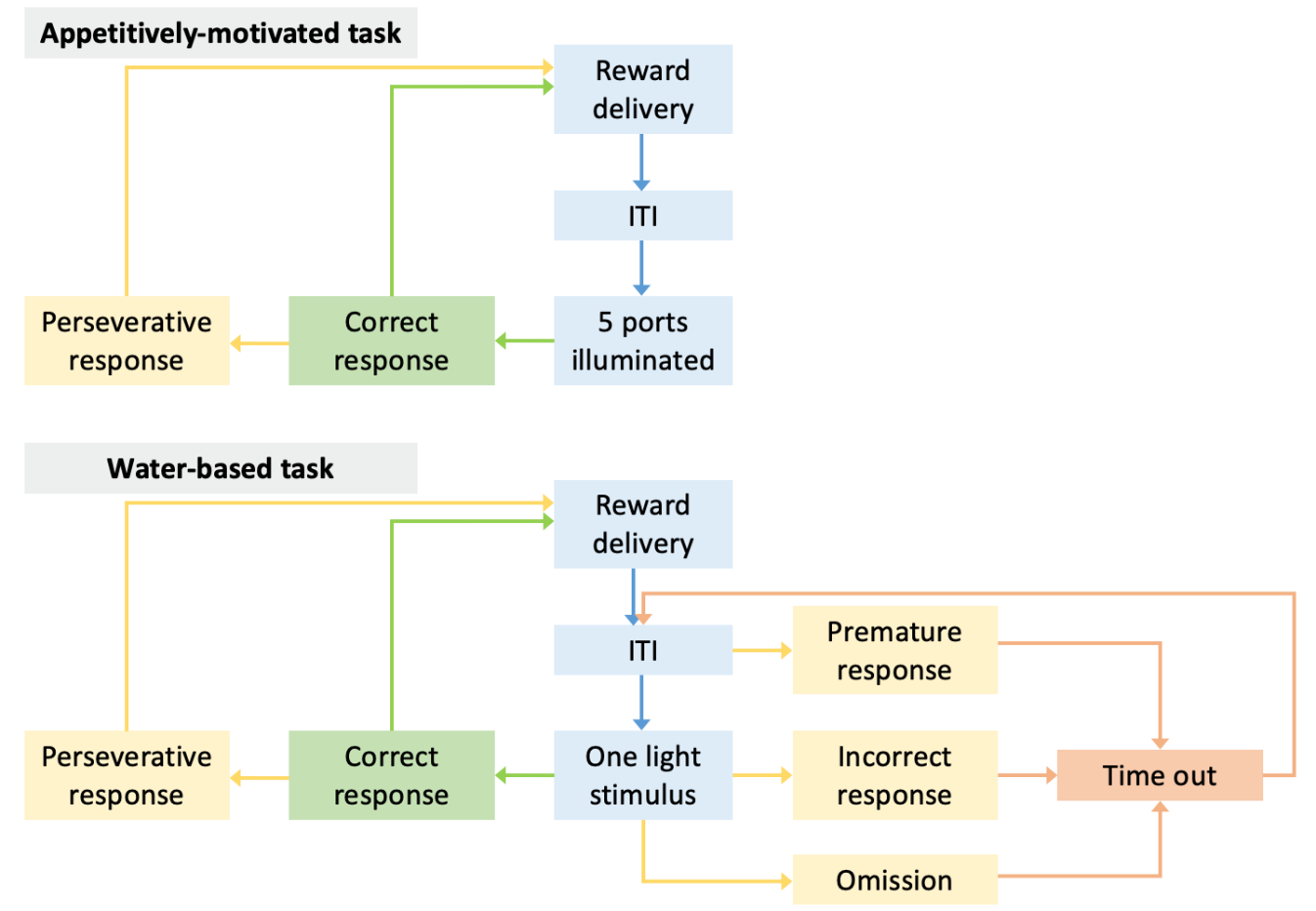


**A**

**B**


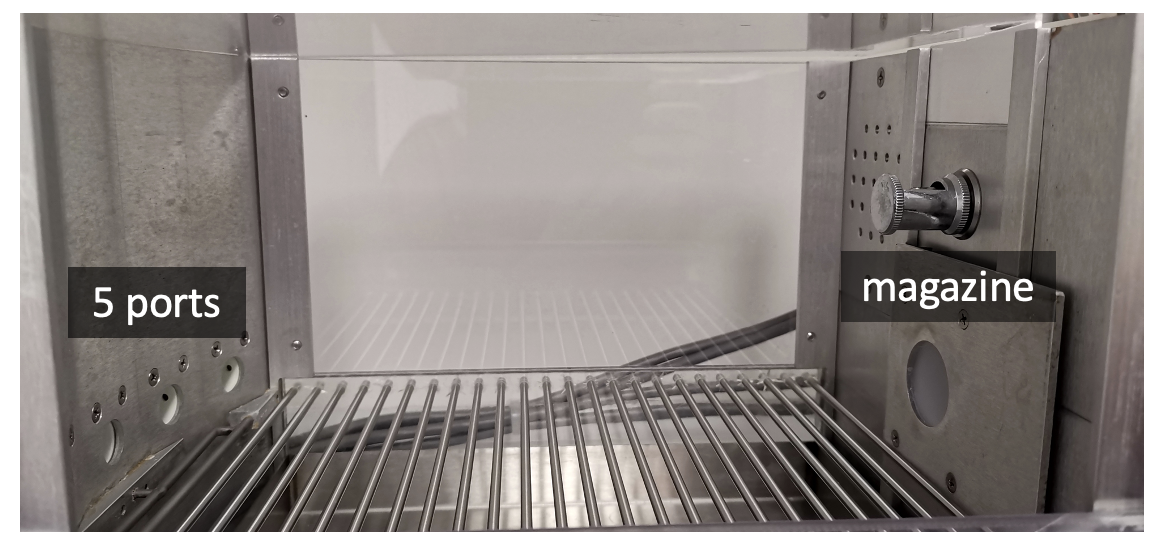


**Supplementary Figure 2 | Photograph of the operant chamber setup together with illustration of the task used.** **(A)** 5-poke operant chamber (Med Associates) and **(B)** task used in the appetitively-motivated operant paradigm.

**B****v**

**A****v**

**C****v**

**Supplementary Figure 3 | Additional data from the social preference test in VGLUT3 cKO^5‑HT^ mice and controls**. **(A)** Time spent in the mouse chamber and **(B)** total distance travelled on the apparatus in male and female VGLUT3 cKO^5‑HT^ mice (n=12) and controls (n=13). **(C)** Raw data of time spent in the 3 chambers. Bars represent mean ± SEM values, with individual values indicated by closed circles. Chance is indicated by the dashed line (33%). Data were analysed with two-way repeated measure ANOVA. ***p<0.001, *p<0.05.


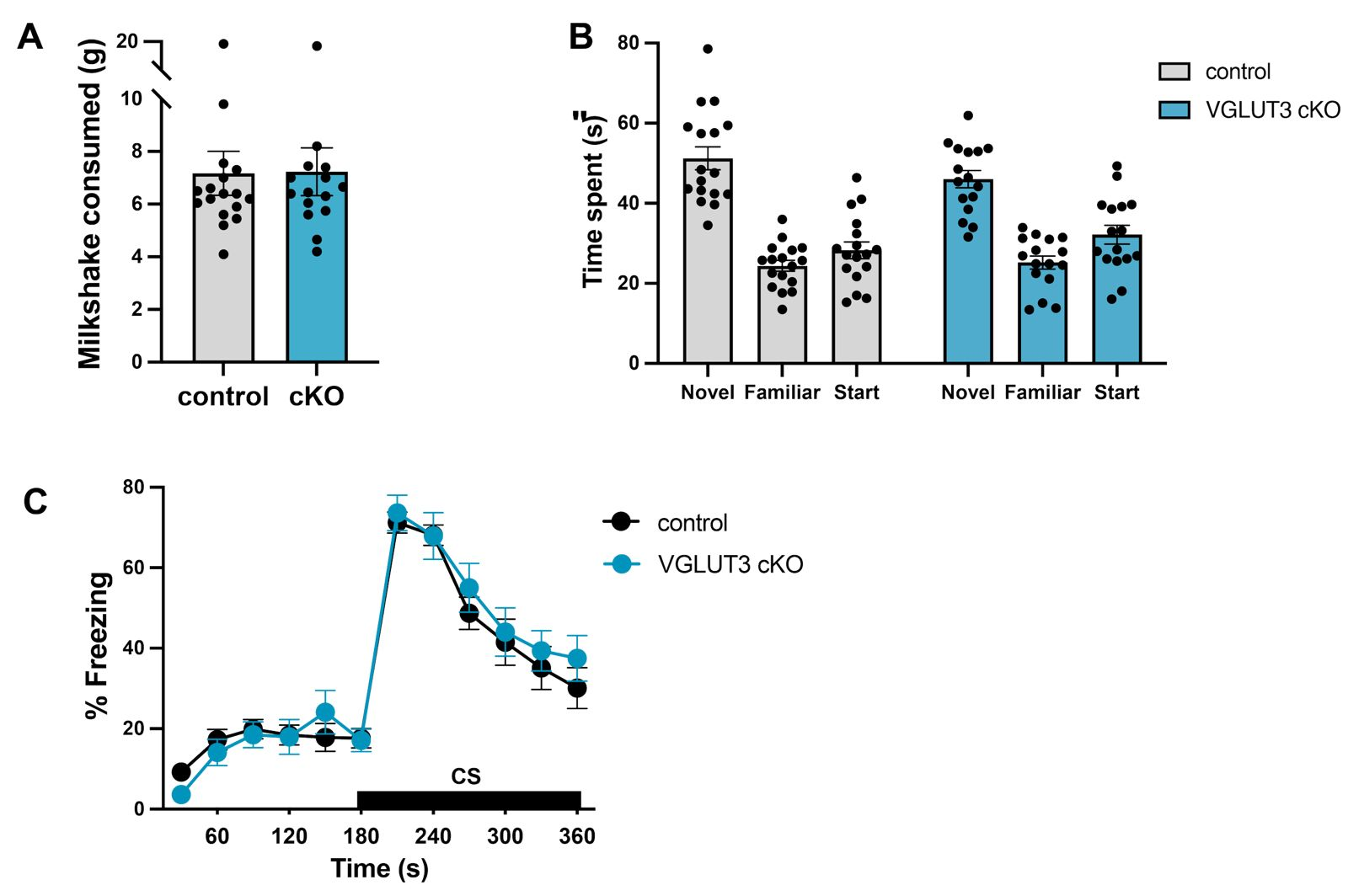


**Supplementary Figure 4 |** **Additional data on milkshake consumption, spatial novelty preference, and cued fear conditioning in VGLUT3 cKO^5‑HT^ mice and controls.** **(A)** Measurements of milkshake consumption in VGLUT3 cKO^5‑HT^ mice (n=15) and control littermates (n=17) during a 4 hr period. **(B)** Raw data of time spent in the 3 arms of the spatial novelty preference test in VGLUT3 cKO^5‑HT^ mice (n=17) and control littermates (n=16). **(C)** Percentage time spent freezing during a cued fear test in VGLUT3 cKO^5‑HT^ mice (n=12) and control (n=8), in 30 s time bins. In (A-B) bars represent mean ± SEM values, with individual values indicated by closed circles, while in (C) closed circles connected with lines, represent mean ± SEM. Data were analysed by t-test or two-way ANOVA.

**
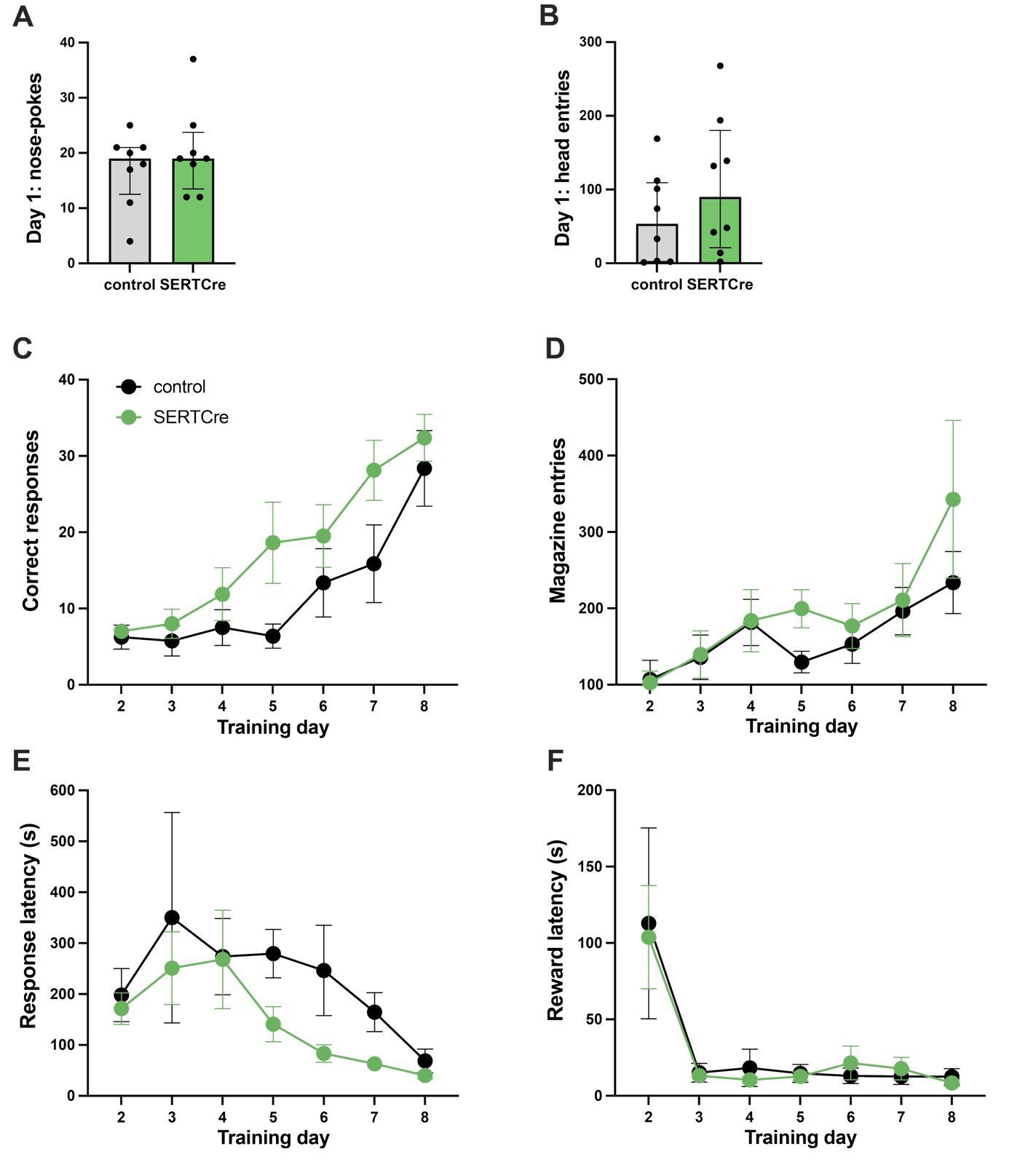
**

**Supplementary Figure 5 | Performance of SERT-Cre mice and controls in the appetitively-motivated operant paradigm**. **(A-B)** Data from day 1 (top row) and **(C-F)** days 2-7 for SERT-Cre mice (n=8) and control littermates (n=8). In (A-B) bars represent mean ± SEM values with individual values indicated by closed circles, while in (C-F) closed circles represent mean ± SEM values (some error bars are too small to be depicted). Data were analysed by t-test or repeated measures mixed-effect model.

**Supplementary Table 2 | Behaviour of SERT-Cre mice compared to control littermates**. Values are mean ± SEM or median [25^th^, 75^th^] values. Analysed by t-test.

| **Test and parameter** | **Control** | **SERT-Cre** |
| --- | --- | --- |
| **EPM** | n=10 | n=9 |
| Open arms entries | 9.7 ± 2.2 | 8.9 ± 0.8 |
| Time in open arms (s) | 78.2 ± 13.0 | 65.6 ± 7.3 |
| Total distance travelled (m) | 12.9 ± 1.3 | 13.6 ± 0.4 |
| **Novelty-induced hyponeophagia** | n=10 | n=13 |
| Latency to contact (s) | 3.5, [2, 6.8] | 5, [3, 7] |
| Latency to drink (s) | 54.3 ± 21.7 | 49.6 ± 9.8 |
| **Sucrose preference test** | n=9 | n=6 |
| Sucrose preference | 1.7 ± 0.2 | 1.3 ± 0.3 |
